# Establishing a Southern Swedish Malignant Melanoma OMICS and biobank clinical capability

**DOI:** 10.1186/2001-1326-2-7

**Published:** 2013-02-27

**Authors:** Charlotte Welinder, Göran Jönsson, Christian Ingvar, Lotta Lundgren, Håkan Olsson, Thomas Breslin, Ákos Végvári, Thomas Laurell, Melinda Rezeli, Bo Jansson, Bo Baldetorp, György Marko-Varga

**Affiliations:** 1 Department of Oncology and Cancer Epidemiology, Clinical Sciences, Lund University and Skåne University Hospital, Lund 221 85, Sweden; 2 Department of Surgery, Clinical Sciences, Lund University, SUS, Lund 221 85, Sweden; 3 Clinical Protein Science & Imaging, Biomedical Center, Dept. of Measurement Technology and Industrial Electrical Engineering, Lund University, BMC C13, Lund 221 84, Sweden; 4 BioInvent Int. AB, Sölvegatan 41, Lund SE-223 70, Sweden; 5 First Department of Surgery, Tokyo Medical University, 6-7-1 Nishishinjiku Shinjiku-ku, Tokyo 160-0023, Japan

**Keywords:** Malignant melanoma, Protein sequencing, Proteomics, Genes, Antibodies, mRNA, Mass spectrometry, Bioinformatics

## Abstract

**Background:**

The objectives and goals of the Southern Swedish Malignant Melanoma (SSMM) are to develop, build and utilize cutting edge biobanks and OMICS platforms to better understand disease pathology and drug mechanisms. The SSMM research team is a truly cross-functional group with members from oncology, surgery, bioinformatics, proteomics, and genomics initiatives. Within the research team there are members who daily diagnose patients with suspect melanomas, do follow-ups on malignant melanoma patients and remove primary or metastatic lesions by surgery. This inter-disciplinary clinical patient care ensures a competence build as well as a best practice procedure where the patient benefits.

**Methods:**

Clinical materials from patients before, during and after treatments with clinical end points are being collected. Tissue samples as well as bio-fluid samples such as blood fractions, plasma, serum and whole blood will be archived in 384-high density sample tube formats. Standardized approaches for patient selections, patient sampling, sample-processing and analysis platforms with dedicated protein assays and genomics platforms that will hold value for the research community are used. The patient biobank archives are fully automated with novel ultralow temperature biobank storage units and used as clinical resources.

**Results:**

An IT-infrastructure using a laboratory information management system (LIMS) has been established, that is the key interface for the research teams in order to share and explore data generated within the project. The cross-site data repository in Lund forms the basis for sample processing, together with biological samples in southern Sweden, including blood fractions and tumor tissues. Clinical registries are associated with the biobank materials, including pathology reports on disease diagnosis on the malignant melanoma (MM) patients.

**Conclusions:**

We provide data on the developments of protein profiling and targeted protein assays on isolated melanoma tumors, as well as reference blood standards that is used by the team members in the respective laboratories. These pilot data show biobank access and feasibility of performing quantitative proteomics in MM biobank repositories collected in southern Sweden. The scientific outcomes further strengthen the build of healthcare benefit in the complex challenges of malignant melanoma pathophysiology that is addressed by the novel personalized medicines entering the market.

## Background

Malignant melanoma is a disease that is increasing in the industrialized world. In Sweden approximately 2800 new cases are diagnosed every year and 500 patients die of disseminated melanoma every year. At the time of diagnosis 10-15% of these cases are considered advanced and this late discovery of the disease means a very poor prognosis. For malignant melanoma there is today no curative treatment when the tumor has disseminated, because malignant melanoma is relatively resistant to both chemotherapy and radiotherapy [1]. The prevalence of mortality due to malignant melanoma is increasing and accounts for 4% of all skin cancers. Melanoma is a malignancy of pigment-producing cells (melanocytes) located predominantly in the skin, where it causes the greatest number of skin cancer-related deaths worldwide. The increasing number of elderly people in society, *i.e.*, above 65 years has a huge impact on the healthcare costs, and continues to escalate in the near future. Therefore new developments are essential for hospitals to improve healthcare efficiency as well as provide superior healthcare to patients. The current research developments and future healthcare solutions are expected to be closely linked to the utility of biobank initiatives. A paradigm shift is under development, using future 4P medicines, that is expected to introduce a paradigm shift in medical practice. The 4P Medicine is one of the most innovative future visions, that currently is under development, with the objective to develop: personalized/predictive/preventive/participatory treatments [2].

Global access to biobank samples associated with clinical data, as well as standardizations is key factors in order to serve the patients on a global scale. The number of biobanks is increasing as well as the number of samples that these large archives hold. In order to improve on efficiency and to optimize the sample per biobank-freezer area, which includes robotic handling and processing of individual sample tubes, miniaturization is crucial. The miniaturization is technology driven, which results in less sample consumption for each assay, as well as a decrease in overall blood handling for the hospitals. Accordingly, this has a direct impact on biobank blood storage requirements, where the ultimate goal is to reach lowest cost per sample on a given −80°C storage units [3]. Currently biobank sources are being used more extensively in order to undertake large population based studies, from where new diagnostics and novel drugs can be developed. Biobanking was recently nominated as one out of ten most important ideas changing the world right now [4].

Today we have a lack of reliable diagnostic tools that can identify early malignant melanoma as well as lack of sensitive methods/tools to follow the progression of the disease, monitor the treatment or identify patients in high and low risk groups, respectively. A number of markers associated with malignant melanoma (*e.g.* S-100 and 5-S-cysteinyldopa) are under investigation, but their relevance to melanoma progression, clinical outcome and the selection of optimal treatment strategies still needs to be established [5]. Recently mutations in *B-RAF* (v600E) have been identified as an important driver of malignant melanoma. Inhibitors to *B-RAF* have likewise been shown to very favorably affect melanoma growth. More than 50%of melanoma cases harbor mutations in the gene. In published phase III clinical studies [6,7], the median progression free survival is 6 month mainly due to development of resistance to the treatment. The Department of Oncology at Skåne University Hospital has participated in clinical trials with *B-RAF*-inhibitors. Characterization of the somatic melanoma mutations in *B-RAF* (v600) will lead to a better understanding of developmental pathways of melanoma. Possible targets for melanoma therapy are delineated with relevant inhibitors already in research or on its way out in the clinic. In this project we aim to find new tumor markers for malignant melanoma with an ultimate goal to apply these findings in the clinic, with an ability to investigate drug impact and mechanisms of drug action, as well as getting a better understanding of MM pathophysiology. An ultimate goal for the SSMM is to organize the biobank patient samples in a systematic way by harmonization in order to provide the highest quality of the MM archive [8].

## Experimental

### Clinical material

Metastatic tissue from malignant melanoma cancer patients was obtained from the Skåne University Hospital and other hospitals in the southern part of Sweden. Ethical approval was granted by the Lund University (approval number: DNR 1912007).

### Histology

Cryostat thin slices, 10 μm of thickness, of lymph node metastases from patients with malignant melanoma, stage III, participating in the South Swedish study, were prepared using a Leica CM5030 cryostat. Serial sec-tions were placed upon frosted end microscope slides (Menzel-Gläser) and air-dried followed by incubation at 37°C for 30 min. The slides were then stained with Hematoxylin–Eosin.

### Sample preparation

Proteins were extracted with AllPrep DNA/RNA/Protein Mini Kit (Qiagen) according to manufacture’s instructions. The extracted proteins were precipitated with ice-cold acetone to a final concentration of 80% acetone. Samples were incubated for 30 min at −20°C followed by centrifugation at 16000 *g* for 2 minutes. The supernatant was removed, and the protein pellets were allowed to air dry. The dried protein pellets were resolved in 8 M urea in 50 mM ammonium bicarbonate (pH 7.6). Protein concentration was determined by the BCA method (Pierce). The proteins were reduced with 10 mM dithiolthreitol (1 h at 37°C) and alkylated using 40 mM iodoacetamide (30 min, kept dark at room temperature). Following buffer exchange with 50 mM ammonium bicarbonate buffer (pH 7.6) by using a 10 kDa cut-off spin filter (YM10 filter, AMICON). The samples were digested with sequencing grade trypsin (Promega) in ratio 1:100 w/w (trypsin: protein) overnight at 37°C.

### MRM assay development

Crude peptides were supplied by Thermo Scientific. Three mixtures were created from the crude peptides, ca. 15 peptides in each and one mixture with a concentration of 50 fmol/μL of each peptide. The transition lists were created in Skyline v1.2 software (MacCoss Lab). Primarily, high numbers of transitions, all possible y-ion series that matches the criteria (from *m/z* > precursor-2 to last ion-2, precursor *m/z* exclusion window: 20 Th), were selected for each peptide at both 2+ and 3+ charge states. The peptide mixtures were analyzed by nanoLC-MS/MS using a TSQ Vantage triple quadrupole mass spectrometer equipped with an Easy n-LC II pump (Thermo Scientific, Waltham, MA). The samples were injected onto an Easy C18-A1 pre-column (Thermo Scientific, Waltham, MA), and following on-line desalting and concentration the tryptic peptides were separated on a 75 μm × 150 mm fused silica column packed with ReproSil C18 (3 μm, 120 Å from Dr. Maisch GmbH, Germany). Separations were performed in a 45-min linear gradient from 10 to 35% acetonitrile containing 0.1% formic acid; at the flow rate of 300 nL/min. The MS analysis was conducted in positive ion mode with the spray voltage and declustering potential were set to 1750 V and 0 V, respectively. The transfer capillary temperature was set to 270°C and tuned S-lens value was used. SRM transitions were acquired in Q1 and Q3 operated at unit resolution (0.7 FWHM), the collision gas pressure in Q2 was set to 1.2 mTorr. The cycle time was 2.5 s in the non-scheduled methods and 1.5 s in the scheduled methods. The best transitions (3–5 per precursor) were selected by manual inspection of the data in Skyline and scheduled transition lists were created for the final assays. The selected transitions were tested in real matrix also by spiking the heavy peptide mixtures into human malignant melanoma tissue digests.

## Results and discussion

### Biobanking malignant melanoma

Disease presentation and clinical sample collections are key strategic resources that need to be installed in order to build on a disease understanding that generates the next generation of modern treatments and diagnostics for MM patients. In that respect, there are an increasing number of large-scale population-based studies currently collecting vast numbers of clinical samples into biobank repositories [9]. These sample collections will be the source of much information in the years to come. The UK Biobank has collected blood samples from 500,000 participants in the study, which are stored in −80°C freezers in the UK Biobank in Manchester (http://www.ukbiobank.ac.uk/about-biobank-uk), whereas a Swedish initiative with automated processing and robotic handling and sorting is generating millions of sample tubes over the years [5]. In this context, the human proteome initiative, that will map all of our human proteins, are stakeholders in these biobank establishments [10].

Disease link-related data are one important deliverable that will be of high value and of cardinal importance for generating added values to:

• Disease understanding

• Novel Patient Diagnosis

• New Drug Development

The strategy of the SSMM research group is to harvest the values in the clinical MM materials that are built in our automated large-scale biobank. We will utilize blood fractions as a resource for biomarker and genomics studies as well as tissues from drug treated patients, where the spatial drug distribution in tissue is mapped by imaging mass spectrometry [11,12]. In order to improve on both the efficacy and safety profiles of novel medicines, a detailed understanding of both drug characteristics, and mechanism of drug action is needed. The new generation of targeted personalized medicines are steadily increasing, in healthcare today with good treatment prognosis. The pharmaceutical companies have filled up their production pipelines, perusing clinical studies, and are expected to reach the market with these novel chemical entities.

**Figure 1:**
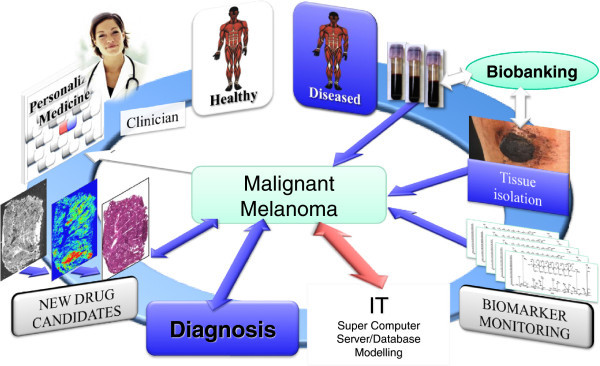
Illustration of the strategy to address malignant melanoma as organized by the Southern Swedish Malignant Melanoma (SSMM) research team.

An improved understanding of genetic heritage and RNA expressions in MM are of fundamental importance in order to discover the genetic factors that can group the patients within MM [13,14]. The correlation with protein expression and disease function is key in order to understand the disease biology in MM. Within the research team there are members who daily diagnose patients with suspect melanomas, do follow-ups on malignant melanoma patients and remove primary or metastatic lesions by surgery. The clinical demographics and registry data will be used in combination with OMICS data, where we will apply bioinformatics to predict MM correlations. The cross-functional approach is depicted in Figure 1, illustrating the interplay in-between the clinical units and the research teams in a collaborative effort.

**Figure 2:**
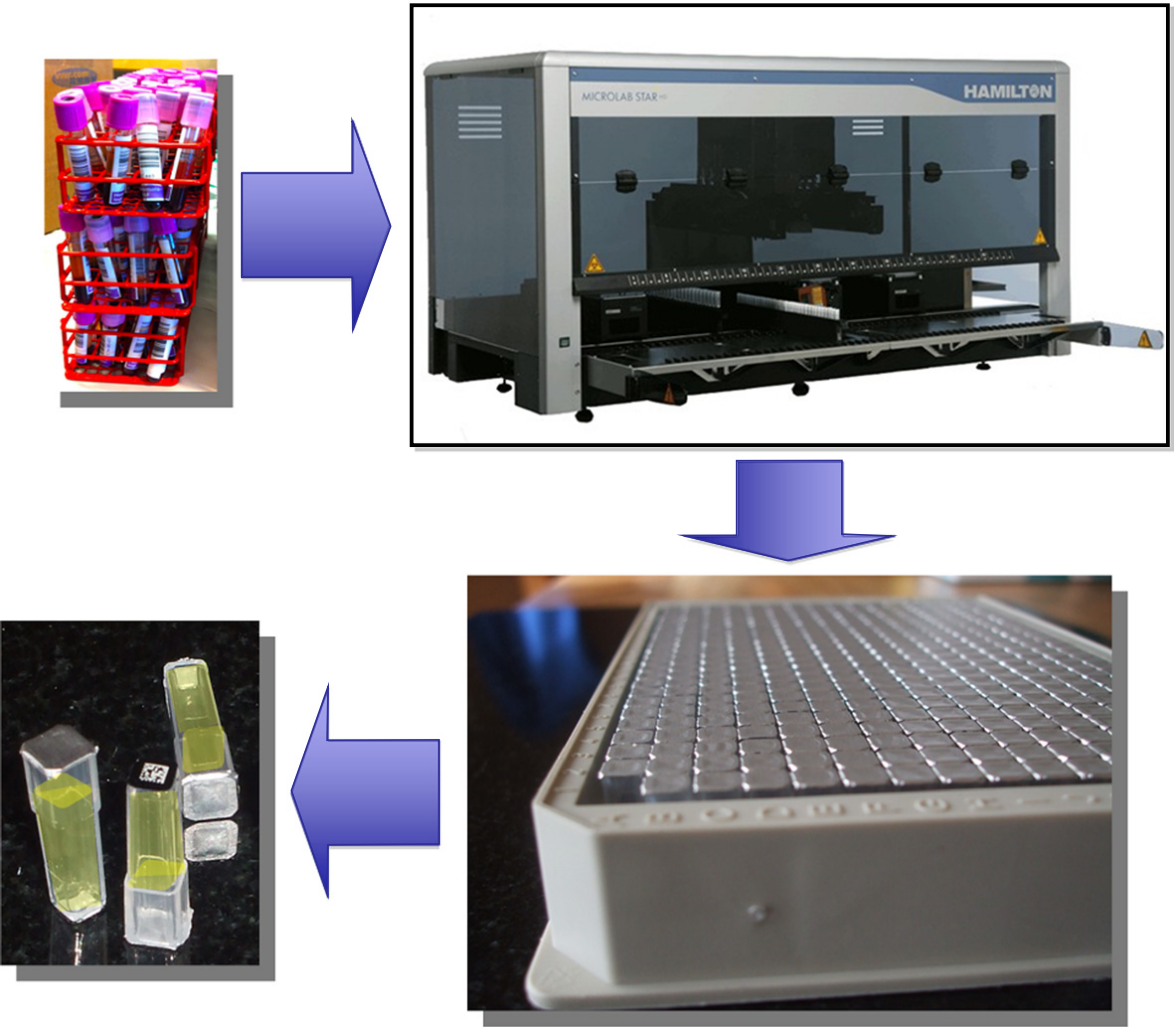
Blood sample processing workflow with 384-tube format storage, providing a complete tracking and 2D bar coded guidance of each individual sample tube within the study.

Our objectives within SSMM are to work integrated to the clinical treatment of MM patients and collect samples as well as clinical data. This is crucial as each sample provides a snapshot of the disease processes in place at the time of sampling [15]. All of these biobank samples will be archived, using standardized protocols and procedures to ensure highest possible quality. As blood is the most common bio-fluid, that can be globally accessed and compared for clinical developments, our strategy for MM biobank developments is to collect both plasma (EDTA, citrate, heparin), as well as serum, and whole blood. The sample processing procedure that will be applied is shown in Figure 2, with the high-density tube system, that was recently validated for Biobank purposes [16].

There is a necessity to use standardized methods for long-term cold storage that should include internal standards to measure spontaneous sample decay over time [17]. Standardization and best practice has reached a global status by the “NCI Best Practices for Biospecimen Resources” document from the NIH in the US (http://biospecimens.cancer.gov/practices/2011bp.asp).

Generally, storage and acquisition of sufficient numbers of study samples might take months or even many years to complete [18,19]. For these reasons sustainable sample processing and its quality assurance over time is mandatory. In this way we will ensure high quality of the patient material that will make the comparative MM studies possible.

For each blood type, forty-eight blood fractions were generated as 70 μL aliquots. All of these processing steps were carefully controlled by the software of the pipetting robot. There is a standard operating procedure (SOP) that is applied to the protocol with built in criteria and quality assurance conditions. The successful completion of handling the primary tubes is followed by the completion of a 384-rack, shown in Figure 2. These steps were controlled by the liquid handling robot, linked and interfaced to the LIMS that provides registered data control of each step processed within the blood fractionation processing.

### Pilot study with a toolbox concept

The sequencing of the human proteome will be performed with a Gene-Centric strategy, where the Gene-Coded Proteins will be addressed, based on previous genomics studies in MM [13,14].

There is a future important ambition of future OMICS research, which relates to determine how the annotated regions of the genome control the production of protein annotations. The research community currently links the Encyclopedia of DNA Elements (ENCODE) consortium that reveals rapid progress in deciphering the human genome with the human proteome [20]. When adequate numbers of samples are stored, analysis will be performed using standardized sample preparation and OMICS analysis with state of the art sample separation protocols. The technology developments within genomics, proteomics and metabolomics are impressively fast. These advancements will drive healthcare efficiency where the human genome map complemented by the map of human proteome will be used as key cornerstones [10].

**Figure 3:**
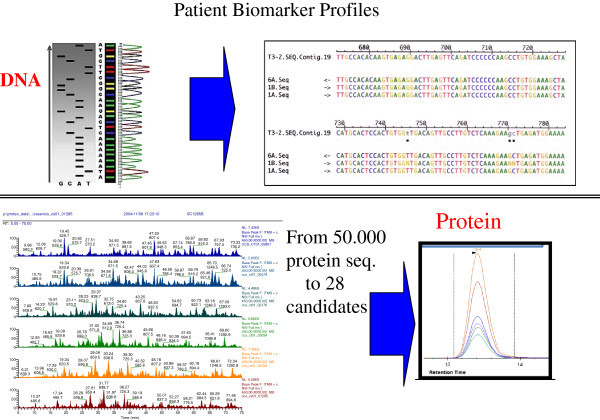
OMICS strategy with genomics screening platforms linked to shotgun sequencing and SRM protein sequence assay platform.

The correlation in-between the human genome, the transcript regulations and the proteins that are being expressed in the human body is key in R&D within clinical sciences, as illustrated in Figure 3. One of our MM strategies is to run: i) Genome mining, ii) Proteome-deep mining and iii) targeted protein screening. Proteins are usually modified after synthesis in the cell, and it is the post-translational active form of the target protein that in most diseases forms the basis for drug developments.

In order to utilize gene-, and proteins as predictors of disease and/or treatments as for drug responders, more experience and R&D inputs will be requested. For diagnostic quantifying of protein(s) in clinical studies, there are currently no guidelines from the Food and Drug Administration (FDA) or European Medicines Agency (EMEA). However, there are ongoing biomarker projects, where the FDA in collaboration with the Academics and pharmaceutical industry are looking into standardization procedures for future utilization. This in contrast to the pharmagenomics area whereas guideline for industry has been introduced recently (http://www.fda.gov/downloads/RegulatoryInformation/Guidances/ucm126957.pdf).

### Protein sequence based assays

There has been an extensive progress made in the last decade within the area of proteomics and protein expression analysis. An increasing number of target and biomarker discovery studies are undertaken in both academia and industry using shotgun sequencing, utilizing mass spectrometry technology [21].

In addition, today most drug targets are being validated by proteomics assays in the pharma and biotech industries, providing evidence to the role of the target within a given disease area.

In retrospect, one of the first reports that was presented on the ability to map a protein expression in human blood plasma, was made with 2D gel electrophoresis with intact proteins, in contrast to the protein digest protocol, followed by LC-MS, that is the most commonly used separation and mass spectrometry platform of today [22]. The marriage and the interface in-between high-resolution chromatographic separation and mass spectrometry have been a critical part of the development of large-scale protein analysis. Today, the mass spectrometer has become the real workhorse for protein sequencing and protein determinations.

### Global expression analysis

In studies where the total protein expression is of interest to build on a disease staging profile, shotgun sequencing is utilized. This is a platform where the proteins are sequenced within a high-throughput scanning cycle. The global expression studies are generally used in “Discovery phases” including a wide scale analysis of many housands of peptide sequences that originates from digested proteins. This global protein expression analysis field is intimately associated with the area of proteomics research. Proteomics of today holds the post genomic research activities, in one big research community, where many of the successes will be expected to be deliver on the understanding of the complex disease pathophysiology. In one respect, the proteomics field has played a significant role in opening up a number of doors that has been important for other researchers, such as the clinical field. The current status is an extensive discovery phase protein candidate delivery that has been reported on by the proteomics society where still the extended validation of candidate proteins, still remains to be pursued.

### Targeted analysis

Targeted protein analysis is dedicated to quantification where a specific and smaller set of proteins is measured in dedicated assays. In the last years, MRM multiplex assay have become very popular due to their generic concept and the ability to generate multiplex quantifications. Currently, protein quantification is preferably performed with an immuno-reagent base assay technique, where there are a large number of methodologies available.

**Figure 4:**
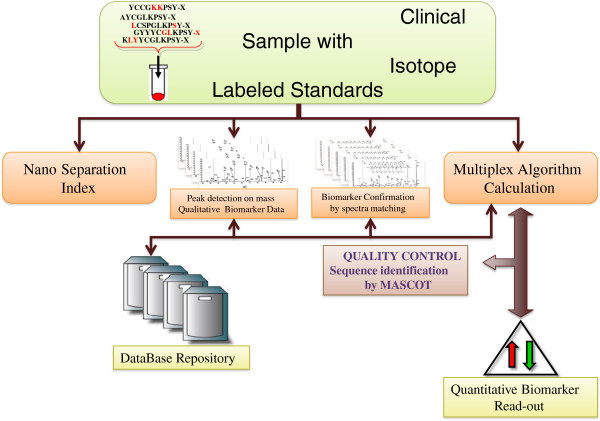
Protein assay development process from Discovery to Targeted MRM assay developments.

Protein biomarkers are identified as differentially expressed in clinical samples comparing for instance clinical status of disease and health. Patients are selected carefully by various clinical criteria, but usually with an emphasis to have clinical data that group the patients in a given disease group that reflect the staging of disease. The discovery output will result in a list of proteins with differential protein expression in relation to a control group. The targeted MRM/SRM mass spectrometry assays, identifies and quantifies specific protein sequences within the sample. MRM/SRM assays offer high-speed analysis, which is a future requirement in high throughput screening of clinical samples for candidate biomarkers. Figure 4 illustrates the basic principles of SRM technology where absolute quantification is reached with multiple proteins in a single assay cycle.

Within SRM or MRM assays, a series of transitions are made with the target peptides that are the precursors, being ionized after the LC-separation and interfaced to MS. Looking back to decades of biological mass spectrometry and the recent decade with proteomics studies, it is obvious that all the generated protein sequence data, compiled and built within databases is a great asset. These experimental reports provide in many instances not only sequences of importance, but also in numerous cases quantitative information regarding a specific biology of clinical relevance. These data has been generated both within Academia, as well as within pharma-, and biotech- industry.

The initial step of an MRM assay development usually relates to applying an *in silico* step, whereby we can use a selection process, followed by blast searching in protein databases where identified proteins in biological samples can verify the utility of target peptides identified as candidates. Precursors and *m/z* of the peptide products are keys to the assay development and should be identify at an early stage. Best practice for rapid assay developments with MRM-MS instrumentations was recently presented [23]. In this respect, peptide libraries used as standards, are invaluable tools in order to funnel the large number of peptide candidates in the *in silico* processing step that helps make judgments of the most useful target peptide candidates for the assay [24,25].

**Figure 5:**
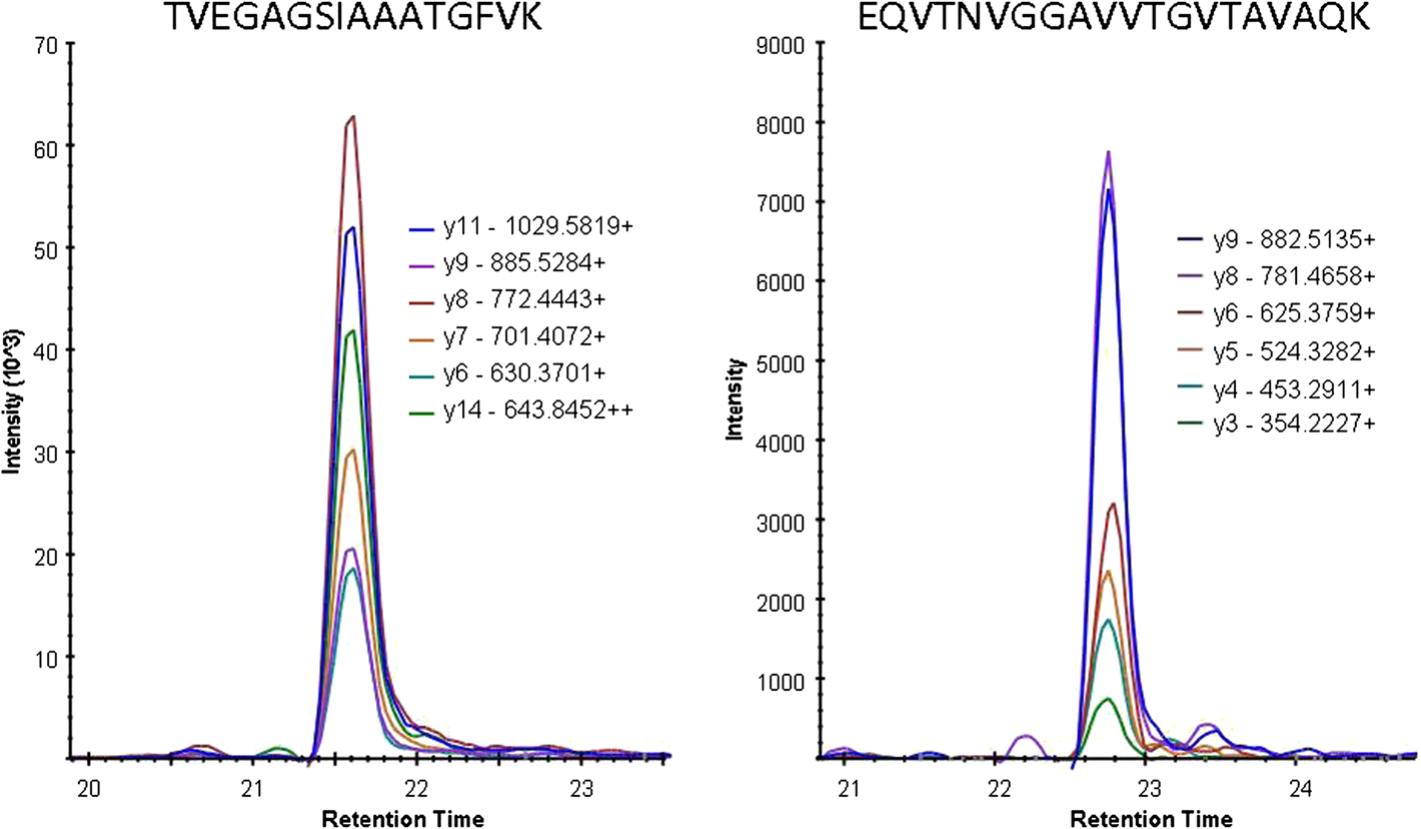
**Resulting mass spectrometry SRM-spectra from lymph node metastasis isolated from MM patients.** SRM assay is targeting two peptides from α-synuclein: TVEGAGSIAAATGFVK (left spectrum), and EQVTNVGGAVVTGVTAVAQK (right spectrum).

Figure 5 provides assay read-outs from a α-synuclein SRM assay, where we have targeted 2 specific sequences of the protein. The respective transition of the resulting peptide ions formed in the assay is shown by a differentiation in colors in Figure 5. Possible tools for early detection of metastasis could be markers, *i.e.,* molecules produced by the tumor, which may be found in the blood, such as α-synuclein.

In order to make progress in the field of malignant melanoma treatment, the process of malignant melanoma spread has to be better understood. Identifying the expression patterns of proteins associated with melanoma progression might bring some clarity and better understanding of the progression of malignant melanoma.

### Malignant melanoma pathophysiology

The cutaneous malignant melanoma is a neoplasm originating from the melanocyte cells that can occur either *de novo* or from a pre-existing lesions; congenital, acquired, or atypical nevus. In addition, non-cutaneous primary sites of melanocytes also include the mucosal epithelium, retinas, and leptomeninges. There are four most common MM subtypes, that can be distinguished by clinical and pathologic growth patterns:

• Superficial Spreading

• Lentigo Maligna

• Nodular

• Acral Lentiginous

There is a multidisciplinary approach, a whole healthcare infrastructure team usually is involved to optimize detection and treatment of this increasingly common cancer, that includes primary care physicians, as well as dermatologists, surgeons, oncologists, immunologists, radiologists, pathologists, and epidemiologists. The most common site for melanoma in men is the upper back; in women, the most common sites are the lower legs and upper back. Five stages of tumor progression have been suggested:

1) Benign melanocytic nevi

2) Melanocytic nevi with architectural and cytologic atypia (dysplastic nevi)

3) Primary malignant melanoma, radial growth phase

4) Primary malignant melanoma, vertical growth phase

5) Metastatic malignant melanoma.

**Figure 6:**
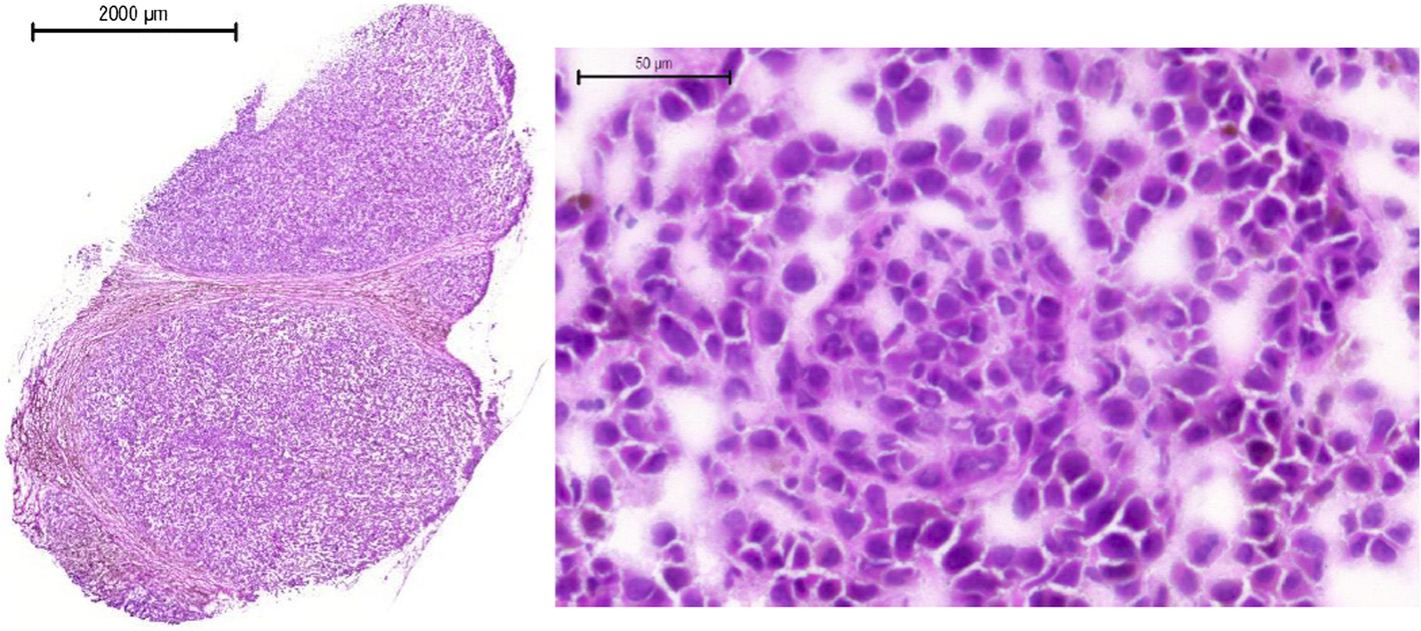
Lymph node metastases from one patient with malignant melanoma, stage III, participating in the southern Swedish study, stained with Hematoxylin–Eosin.

The management for patients who have diagnosed MM occurs with or without a personal or family history of melanoma. The pathologic confirmation of the clinical diagnosis is a key step in patient care and provides a more solid basis for making further management decisions. An example of a surgically removed nodule is shown within the image in Figure 6. The histology section stained with H&E is a typical example within the SSMM patient cohort, and is part of the experimental characterization in order to diagnose the MM patients.

### Data flow & IT-solutions

Within the consortium we have developed an IT-infrastructure, utilizing FDA approved software for sample and data processing, as well as the link to clinical proteomic units at three hospitals/universities, and with national biobank units.

**Figure 7:**
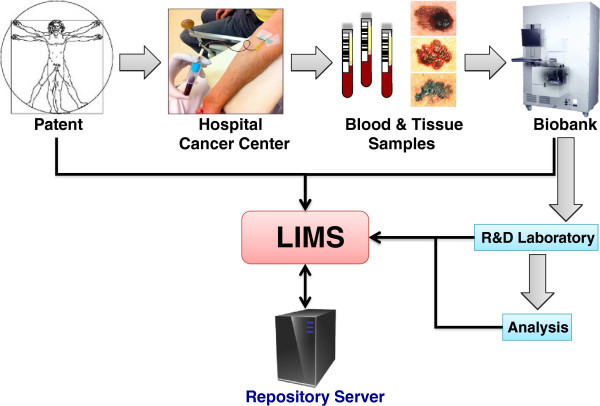
**Schematic illustration of the Southern Swedish Malignant Melanoma work flow where the patients are sampled for blood and/or going through surgery.** These blood samples are processed and aliquoted where after they are stored in the biobank archive at −80°C. Each sample is registered and tracked in the laboratory intelligence management system (LIMS).

The software network and links in-between the MM research teams are depicted in Figure 7 that ensure an efficient and real-time data processing capability. This capability also provides a scheme of the typical workflow within the Southern Swedish Malignant Melanoma research team. MM patients enter into the hospital and are ruled into the biobank collection where both blood fractions and tissue samples are sampled. These samples are processed and stored in 384-tube formats and entered into the bio-storage repository by robotic processing. Resulting analysis data from genomic and protein sequence assays are linked to the 2D barcode of the sample, which is coupled to the patient within the study. The MM database captures all samples as well as the corresponding data, and can be reached by MM consortia members anywhere in the world.

### Mass spectrometry imaging (MSI)

Drug characterization is an ever-increasing research area that currently holds a central position in both drug developments as well as within hospitals where patients are being diagnosed. Classical X-ray is accompanied with MRI, PET and CT for many of the most common diseases including MM.

Mass spectrometry imaging (MSI) is a rapidly developing technique that provides data on the spatial distribution of molecular profiles on a tissue sample. In terms of MM, the MSI can be used to provide evidence of drug presence in tissue microenvironments.

The distributions of drug and metabolite(s) are measured directly in patient tissue samples with a resolving power of 30 μm [26]. This technology platform enables direct drug measurement where pharmaceuticals and metabolites can be analyzed, as a cold compound, without any labeling. It has been gradually accepted that MSI provides rich molecular information provided by this mass spectrometry imaging platform. It interfaces well with general histology, as the staining techniques of tissues are compatible with MSI. This will provide complementary information, and thus offers new possibilities in the clinical field. This ranges from new insights into the molecular changes associated with pathogenesis.

## Conclusions

The Southern Swedish Malignant Melanoma research initiative is a stepping-stone in bridging the bioanalytical and clinical gap, providing an integrated platform and work flow in order to build knowledge and understanding of the molecular and environmental basis of human diseases. The ultimate goals that these large and recent investments into biobank initiatives are striving at are to provide a basis for improving both diagnosis and treatment of disease in society. The research-, and healthcare- societies will also need to support the development of integrated informatics systems that allows for search routines on sample collections, and types, as well as data sets that has been generated from these cohorts. In the end of the day, both the Human Chromosome Initiative, as well as other global research activities are looking for tools that can bridge the data and deliverables that will be of major use in future drug development and patient treatments. Unifying the different disciplines will aid in the fulfilling of the big picture that some of us call personalized medicine and systems biology.

The Human Proteome Project (HPP), which is a global initiative, will have an important contribution by mapping all proteins coded by the human genome. This proteome initiative will provide public access to experimental data on the human proteome, with annotations made in clinical material and in a large number of human diseases. The purpose of this large initiative is to increase the knowledge regarding the identification of disease markers, potential targets of therapy, as well as the insight into variations of expression observed during development, health, and disease (http://www.HUPO.org).

## Abbreviations

SSMM: Southern swedish malignant melanoma; MRM: Multiple reaction monitoring; MM: Malignant melanoma; LC: Liquid chromatography; MS/MS: Tandem mass spectrometry; SRM: Selected reaction monitoring; FDA: Food and drug Administration; MSI: Mass spectrometry imaging; ACN: Acetonitrile; LIMS: Laboratory intelligence management system; HPP: The human proteome project

## Competing interests

The authors declare that they have no competing interests.
